# Bioactivities of Flavonoids from* Lopezia racemosa*

**DOI:** 10.1155/2019/3286489

**Published:** 2019-04-11

**Authors:** Enrique Vergara Barragán, Horacio Bach, Socorro Meza-Reyes, Sara Montiel-Smith, Eugenio Sánchez-Arreola, Luis Ricardo Hernández

**Affiliations:** ^1^Facultad de Ciencias Químicas, Benemérita Universidad Autónoma de Puebla, Mexico; ^2^Faculty of Medicine, Department of Infectious Diseases, University of British Columbia, Vancouver, BC, Canada; ^3^Departamento de Ciencias Químico Biológicas, Universidad de las Américas Puebla, Mexico

## Abstract

*Lopezia racemosa *Cav. (Onagraceae) has been used in Mexican traditional medicine to alleviate stomachache, biliary colic, urine retention, stomach cancer, and skin, dental, buccal, and urinary infections. The objective of this study was to determine the bioactivities of specific parts of the plant to scientifically confirm its traditional use. Aerial parts and flowers were macerated and subsequently extracted with hexane, chloroform, and methanol. This study was focused on the analysis of polar components, and thus the methanolic fractions were selected for further investigations. These fractions were evaluated for their antimicrobial activity using a panel of bacterial Gram-positive and -negative strains, as well as fungal strains, including filamentous fungi and yeasts. In addition, the cytotoxic activity of the extract was assessed by MTT using the human-derived monocytic THP-1 and the normal human fibroblast cell lines. Various fractions showed antimicrobial activity against various pathogens, although the most relevant were against* Pseudomonas aeruginosa* and* Trichophyton mentagrophytes*. No inhibition of yeasts was recorded. Only four fractions showed cytotoxic effects when the human-derived THP-1 and fibroblast cells were assessed. The four flavonoids isolated from the extract were luteolin, luteolin-6-C-hexoside, luteolin-8-C-hexoside, and hyperoside. The biological activities presented in this study validate some traditional uses of the plant.

## 1. Introduction

The genus* Lopezia* (Onagraceae) consists of 45 species (www.theplantlist.org) restricted to a large extent to Mexico [1]. The common names of* Lopezia racemosa* (synonym:* L. mexicana*) are “perilla”, “guayabilla”, “brad”, and “cancer weed” [2]. The leaves of* L. racemosa* are used as an infusion to reduce toothache [3], to relieve stomach and throat pain, against stomach cancer, against infections in the urinary tract, to wash skin infections with pus [4], and against diarrhea [5].

Studies of genus* Lopezia* are very scarce and only a previous study from our research group reported the antimicrobial activity of the chloroform and methanolic fractions from* L. racemosa *[6]. In that study, antibacterial and antifungal activities against a panel of strains were reported. Also, the cytotoxic activities of the hexane, chloroform, and methanol extracts against THP-1 cells were reported with IC_50_ of 31 *μ*g/mL, 29 *μ*g/mL, and 30 *μ*g/mL, respectively [6].

Another study reported the production of the anti-inflammatory compound 6-O-palmitoyl-3-O-*β*-D-glucopyranosylcampesterol using a callus culture of the same plant [7].

Following our research program of studying Mexican plants used in traditional medicine, we investigated the methanol extract and its fractions from the aerial parts of* L. racemosa* to evaluate the antimicrobial, antifungal, and cytotoxic activities. The phytochemistry of the fractions was also studied and the identified compounds were flavonoids.

## 2. Material and Methods

### 2.1. Chemicals

The following reagents were used: hexane, chloroform, methanol, and acetone from RBM (Puebla, Mexico); dimethyl sulfoxide (DMSO), formamide (FA), HPLC-grade methanol, H_3_PO_4_, HCl, AlCl_3_, Na acetate, H_3_BO_3_ were purchased from Sigma-Aldrich.

### 2.2. Strains and Culture Media

The Gram-negative strains used in this study were* Acinetobacter baumannii* (ATCC BAA-747),* Escherichia coli* (ATCC 25922),* Pseudomonas aeruginosa* (ATCC 14210), and* Salmonella typhimurium* (ATCC 13311). The Gram-positive strains were* Bacillus subtilis* (ATCC 6633),* Staphylococcus aureus* (ATCC 25923), methicillin-resistant* Staphylococcus aureus* (MRSA) (ATCC 700698), and* Streptococcus pyogenes* (ATCC 51878). The filamentous fungi included* Aspergillus fumigatus* (ATCC 1022) and* Trichophyton mentagrophytes* (ATCC 9533), whereas* Candida albicans* (ATCC 10231) and* Cryptococcus neoformans* var.* grubii* (provided by Dr. Karen Bartlett, University of British Columbia, BC, Canada) represented pathogenic fungi. Bacterial strains were cultured at 37°C in Mueller-Hinton broth (B&D), whereas fungal strains were cultured at 28°C using Sabouraud broth (B&D).

### 2.3. Plant Material and Extract Preparation

*L. racemosa* was collected in San Francisco Papalotla, Tlaxcala, Mexico, at 2,000 m above sea level (19°10'02.2”N, 98°11'37.1”W). A voucher specimen numbered 59890 was deposited in the Herbarium of the Benemérita Universidad Autónoma de Puebla, México. The plant was identified by the Lic. Allen James Coombes.

500 g of dried leaves was successively macerated with 5 L of hexane, 6.5 L of chloroform, and 7.5 L of methanol for 24 h under stirring, obtaining 13.2 g (2.64%), 11.72 g (2.34%), and 53.21 g (10.64%) of extract, respectively. The solvents were evaporated under reduced pressure to dryness using a rotary evaporator (Büchi, Switzerland). A column packed with Diaion HP-20 (60 cm x 10 cm) (Mitsubishi Chemical Corp., Japan) was used to apply 20 g of the methanol extract. Compounds were eluted with a water-methanol polarity gradient from 100:0 to 0:100 and the separation was monitored by UV spectroscopy.

A similar protocol was followed for compound extraction using collected flowers, but using a total of 50 g of dried flowers and 500 mL of each solvent.

### 2.4. HPLC Purification

The separation of the compounds was performed with an HPLC instrument (Agilent 1200) equipped with a photodiode array detector (G1315B), an autosampler (G1329B), and Agilent Chemstation software. Chromatographic separation was performed using an Econosil C18 column (250 x 10 mm, particle size 10 *μ*m, Alltech). A solvent gradient was run starting from 60:40 methanol-H_3_PO_4_ (50 mM) during 10 min and then 50:50 methanol-H_3_PO_4_ (50 mM) for 15 min, 90:10 methanol-H_3_PO_4_ (50 mM) for 25 min, and finally 100% methanol for 30 min. The separation was performed at room temperature (24°C) and the flow rate was 1 mL/min. The injection volume was 100 *μ*L and the total run time was 60 min. The detection of compounds was made at 240, 250, 260, 270, 280, 290, 300, and 360 nm. Samples were prepared by dissolving 5 mg of the fraction in 100 *μ*L of the appropriate solvent (MeOH, DMSO or FA). Compounds** 1**,** 2**, and** 3 **were isolated from fractions 216 – 229 (eluted using 30% water), 274 – 278, and 279 – 293 (both eluted using 20% water) with amounts < 0.7 mg, respectively. Compound** 4** (1.1 mg) was isolated from fractions 744 – 749.

### 2.5. Chemical Structure Assignment

The mass fragmentations of the isolated compounds were determined by fast atom bombardment (FAB) and electrospray ionization (ESI). In the case of FAB, a JEOL instrument equipped with JMS-700D Mstation was used. The running was performed using a FAB^+^ mode with Xe (ionizing gas), an ionization temperature of 29°C, an energy of 6 keV, and an emission current of 6 mA.

Regarding the ESI experiment, an Advion Expression S CMS was used with N_2_ as the ionizing gas and direct injection of the sample. The instrument was run in negative and positive polarities. In the case of the negative polarity, the capillary temperature was at 200°C and the capillary voltage was 180 V with a voltage source of 30 V. The ESI source used a gas temperature of 250°C and a voltage of 2,500 V. The positive polarity used a capillary temperature of 250°C, a capillary voltage of 180°C, and a voltage source of 20 V. The ESI source used a gas temperature of 250°C and a voltage of 3,150 V.

The identification of the compounds was performed by UV spectroscopy (UV-VIS Varian Cary-1) using quartz cuvettes and according to published methodology [8]. The compound identification was performed according to displacement reagents and comparison to published spectra [8]. NMR experiments were performed in a Bruker Avance III 400 MHZ equipment.

### 2.6. Biological Assays

Because of the high polarity of the isolated compounds, DMSO and FA were used as the dissolving agents. As a result of the toxicity of both solvents, the final concentration in the culture of bacteria and fungi was 1% and 5% for FA and DMSO, respectively. When the THP-1 and fibroblast cell lines were assessed, the final concentration of DMSO in the culture was 1%. Fractions dissolved in FA were not assessed as the final concentration of FA in the culture >0.1% was lethal for the cells.

#### 2.6.1. Antibacterial Assay (MIC)

The antibacterial assay was performed as published [6]. Briefly, in a 96-well plate, a mixture of compound and inoculum in 100 *μ*L Mueller-Hinton broth was dispensed. Samples were tested in the following concentrations: 25, 50, 100, 125, 250, and 500 *μ*g/mL. The inoculum was prepared to have a final optical density of 0.05 _*λ*=600_. The mixtures were incubated in a shaker at 37°C for 18 h. The MIC was determined as the concentration of the compound at which no bacterial growth was observed.

#### 2.6.2. Antifungal Assay

The antifungal assay was very similar to the antibacterial assay with some minor modifications. Fungal strains were cultured in Sabouraud broth at 30°C for 24-48 h or until the untreated control showed growth. The yeast inoculum was prepared as in the previous section, while the filamentous fungi were grown from spores as published [9].

#### 2.6.3. Cytotoxicity

The analysis of the cytotoxicity of the compounds was performed using the human-derived monocytic THP-1 (ATCC TIB-202) and normal fibroblast (PromoCell, C-12352) cell lines. The assay was performed as previously published [6]. Both cell lines were treated with the compounds and untreated cells and DMSO were used as negative control, whereas 2% Tween-20 was used as a positive control. The cytotoxicity was determined by the MTT assay [10]. The fractions were tested at a final concentration of 20 *μ*g/mL (DMSO<1%).

### 2.7. Statistical Analysis

A Student's t-test was used to assess the significance of the cytotoxicity. Experiments were performed in triplicate.

## 3. Results

A total of 961 and 465 fractions were obtained from the methanol extracts of leaves and flowers, respectively. These fractions were subsequently grouped in 117 and 85 fractions according to their UV profile, respectively.

The methanol extract was mainly composed of a complex mixture of tannins and flavonoids, which were difficult to purify. Therefore, a few fractions with the highest content of compounds were used to separate and identify the proposed structures, which were based on the UV-Vis behavior using displacement reagents and verified according to published spectra [8].

### 3.1. Chemical Structure Assignment

The methanolic fraction 216-229 from leaves showed a characteristic UV spectrum of a flavone because of the maximal absorption of the band I at 352 nm. In addition, the band II showed two maximal absorptions at 266 (band IIa) and 256 nm (band IIb), indicating the presence of two hydroxyl groups at positions 3' and 4' [8]. A chemical shift was observed when NaOMe was added, which produced a bathochromic shift of 52 nm in the band I without decrease of the intensity or the breakdown of the molecule after 5 min after addition, confirming the presence of a 4'-OH group and absence of the 3-OH group. In addition, a bathochromic shift of 16 nm was observed in band II after the addition of NaOAc, which indicated the presence of the 7-OH group after the stability of the molecule was confirmed. Sequential addition of H_3_BO_3_ to the same reaction caused a bathochromic shift of 19 nm in the band I, confirming the presence of an ortho-dihydroxylic system in the ring B. Moreover, the addition of AlCl_3_ caused a bathochromic shift of 72 nm in the band I, with a posterior hypsochromic shift of 25 nm after the addition of HCl. These changes in the bands confirmed again the presence of an ortho-dihydroxylic system in ring B together with the presence of the 5-OH group.

**Figure 1:**
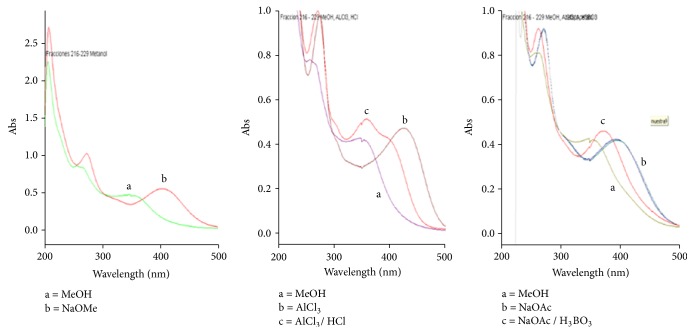
UV absorption spectra of the methanolic fractions 216 – 229 in MeOH and shift observed after the addition of displacement reagents.

**Figure 2:**
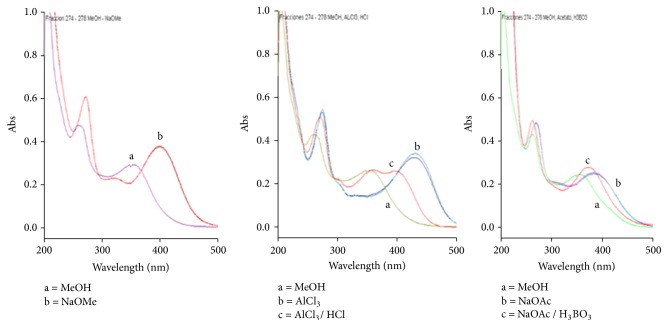
UV absorption spectra of the methanolic fractions 274 – 278 in MeOH and shift observed after the addition of displacement reagents.

**Figure 3:**
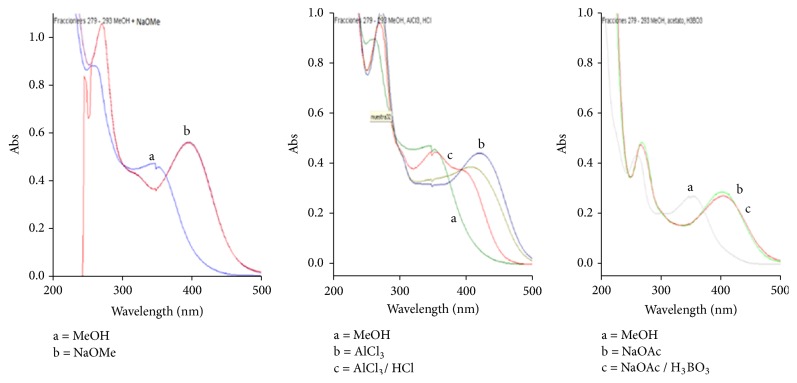
UV absorption spectra of the methanolic fractions 279 – 293 in MeOH and shift observed after the addition of displacement reagents.

After a comparison of the spectra obtained (Figure 1) with the spectra published by Mabry [8], we conclude that the compound** 1** has the luteolin-6-C-hexoside structure, with spectra comparable to the compound isoorientin. Following the same procedure, the flavonoids present in the fractions 274 – 278 and 279 – 293 were luteolin (**2**) and luteolin-8-C-hexoside (**3**), respectively, showing comparable spectra to luteolin (Figure 2) and orientin (Figure 3), respectively. We were not able to identify the sugar moiety in compounds** 1** and** 3** because of the very low amount isolated of these compounds.

**Figure 4:**
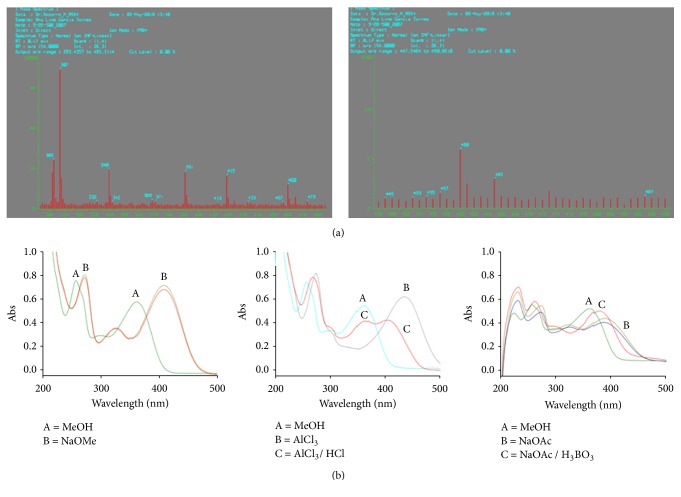
(a) FAB^+^ mass spectrum of** 4**, showing the [M+Na]^+^ (*m/z* 487), the [M+H]^+^ (*m/z* 465), [quercetin+H]^+^ (*m/z *303), and the base peak [quercetin+Na-H_2_O]^+^. (b) UV absorption spectra of** 4** in MeOH and shift observed after the addition of displacement reagents.

The mass spectra of compound** 4** showed the molecular ions [M+H]^+^* m/z* 465 using FAB^+^ and [M+Na]^+^* m/z* 487 and [M+K]^+^* m/z* 503, using ESI^+^. A peak at* m/z* 303 (by FAB^+^) corresponding to the protonated quercetin aglycone was also identified by ESI^+^. Further analysis of the results showed that the ion [M+Na]^+^* m/z* 487 showed a base peak at* m/z* 307, which corresponds to the fragment [quercetin+Na-H_2_O]^+^ (Figure 4(a)). These data concord with the displacement shifts observed in the UV spectra following the techniques previously described (Figure 4(b)). Thus, based on all of the analysis described, it can be deduced that the structure of compound** 4** corresponds to quercetin-3-O-hexoside. NMR experiments confirmed the above findings showing signals in both ^1^H and ^13^C spectra corresponding to the aglycone quercetin. Spectra also showed a glycoside moiety linked to C-3 of the aglycone, which was deduced from the HMBC experiment. The sugar moiety was identified as a galactoside from its ^13^C NMR spectrum. Hence, compound** 4** was identified as hyperoside, which was further confirmed by comparing its spectral data with those published in the literature [11].

**Figure 5:**
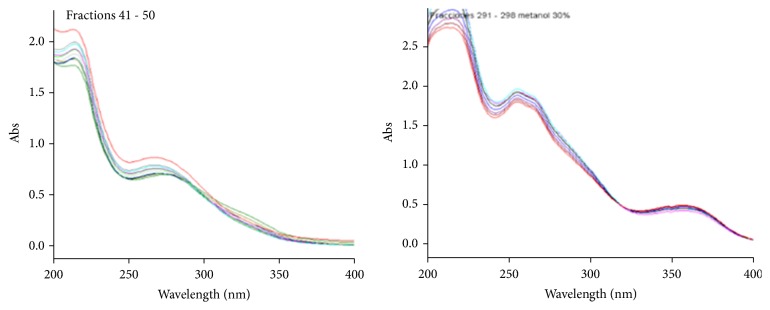
Elution of the methanol extract from flowers separated on a Diaion HP-20.

**Figure 6:**
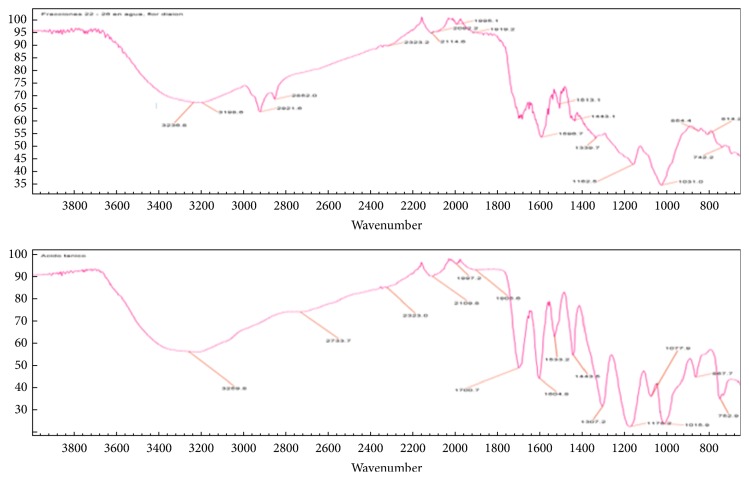
FTIR spectrum of fractions 22 – 26 obtained from the methanolic extract of flowers separated on a Diaion HP-20 (top image) and FTIR spectrum of tannic acid (bottom image).

The fractions obtained from the flower extract showed two types of UV profiles. The first type suggested the presence of tannins in the first fractions, whereas a flavonoid was observed in the last fractions (Figure 5). The IR spectrum of the fraction 22 – 26 was similar to the IR spectrum of the tannic acid (Figure 6), suggesting that this compound can be present in this fraction.

### 3.2. Antimicrobial Assays

Analysis of the different fractions against a panel of bacteria and fungi showed antimicrobial activity against Gram-positive, Gram-negative, and filamentous fungal strains (Table 1). Although most of the fractions gave a relative weak antimicrobial activity (range: 250-500 *μ*g/mL) only the leaf fractions 447-506 (TM=125 *μ*g/mL), 852-856 (PA=50 *μ*g/mL) as well as the flower fraction 304-310 (PA=100 *μ*g/mL) showed antimicrobial activity in the range of potential discovery of pure antimicrobial agents (~100 *μ*g/mL).

### 3.3. Cytotoxicity

**Figure 7:**
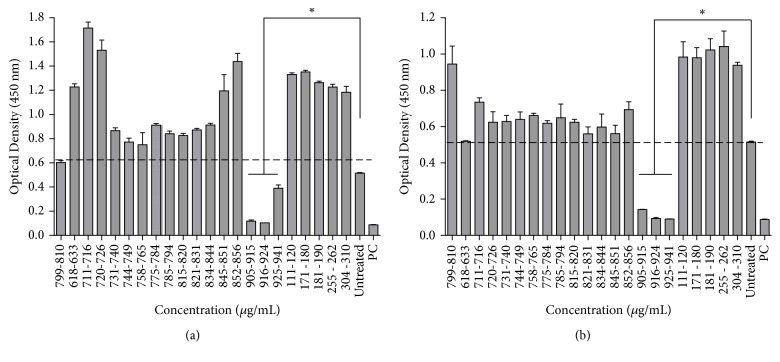
Cytotoxic activity of the methanolic fractions in (a) normal fibroblast and (b) THP-1 cells. PC = positive control. The dash line represents the untreated cells (negative control. Experiments were performed in triplicate. Data are presented as the mean ± SE. *∗* represents the significance level (P<0.05).

Analysis of the cytotoxicity of the fractions showed that most of them were innocuous for both cell lines except the fractions 905-915, 916-924, and 925-941 (Figure 7).

## 4. Discussion

The plant* L. racemosa* is used in the Mexican traditional medicine to treat different diseases, including infections associated with the skin and the urinary system.

In this study, the methanolic extract from leaves and flowers as well as their fractions was assessed for their antimicrobial activities against a panel of Gram-positive, Gram-negative, and fungal pathogenic strains. Analysis of the results showed antibacterial and antifungal activities of 50-500 *μ*g/mL and 125-500 *μ*g/mL, respectively (Table 1) with the strongest activity observed against* P. aeruginosa* and* T. mentagrophytes*. Similar results regarding the antibacterial activity with MICs ranging between 40 and 400 *μ*g/mL were reported in a previous study from our lab [6]. However, the fungal pathogen* Trichophyton rubrum* was inhibited when a concentration of 40 *μ*g/mL was used, whereas we report here a MIC of 125 *μ*g/mL. The discrepancies in the antimicrobial activities can be attributed to the fact that a different method of purification was used in the present study.

As mentioned earlier, reports about the antimicrobial activity of the genus* Lopezia* are rare and thus our results cannot be compared to the literature.

Results of our studies addressed the control of important pathogens such as* P. aeruginosa*, which is a known pathogen causing skin and urinary infections, including complications in burnt skin [12–14]. These infections can be aggravated by the establishment of biofilms [15, 16] with difficulties to eradicate the pathogen.

Another pathogen controlled by the fractions of the plants was* T. mentagrophytes* responsible for skin infections and cutaneous mycosis, including tinea capitis (head), tinea corporis (body), and tinea pedis (foot) [17, 18].

Other skin-related pathogens to which a weak antibacterial activity was measured were* S. aureus*, MRSA, and* S. epidermidis* (Table 1), all of them causing skin infections [19–22], and tonsils, retropharyngeal, and parapharyngeal abscesses [23–25].

A phytochemical analysis of some of the fractions identified the four flavonoids luteolin, luteolin-6-C-hexoside, luteolin-8-C-hexoside, and hyperoside.

In the case of luteolin, antibacterial activities against* P. aeruginosa*,* S. aureus*, streptococcal strains, and* Phorphyromonas gingivalis* with MICs > 100 *μ*g/mL were reported [26, 27]. Other studies reported antibacterial activities against* P. aeruginosa *(MICs = 200-625 *μ*g/mL),* S. aureus *(MIC = 15 *μ*g/mL), and* S. enterica *serovar Thyphimurium (*S.* Thyphimurium, MIC = 20 *μ*g/mL) [28, 29]. The discrepancies between these values can be related either to the purity of the compound or to the strains used to assess the antimicrobial activity.

In the present study, we were not able to identify the sugar group attached to the flavonoids** 1** and** 3** as a result of the very low amount obtained from the isolated compound, except for hyperoside. Frequently, the identification of the sugar moiety is not provided and thus defined as pentoside or hexoside as published [30–32]. Therefore, we discuss the alternative optional names when the monosaccharide of luteolin-6-C-hexoside and luteolin-8-C-hexoside is glucose and then defined as isoorientin and orientin, respectively. In addition, when quercetin-3-O-hexoside contains a glucose unit it is defined as isoquercitrin, a rhamnose unit defines it as quercitrin, and finally, the presence of a galactose unit is defined as hyperoside.

In the case of isoorientin, studies have reported MICs ranging between 100 and 1000 *μ*g/mL for* P. aeruginosa* and* S. aureus*, respectively [26, 28]. In the case of orientin, similar antibacterial activity was reported against* P. aeruginosa* and* S. aureus* as compared to isoorientin [26]. When the antifungal activity was tested against a panel of fungal pathogens, strong antifungal activity was reported against* T. mentagrophytes* (MIC=8 *μ*g/mL) [33].

Analysis of the bioactivities of isoquercitrin and quercitrin showed that both had similar antibacterial activity against a panel of bacterial phytopathogens and* S. aureus* expressed as zone of inhibition [34]. On the other hand, quercitrin has been reported to inhibit the growth of* S. epidermidis*, an important pathogen in periodontal diseases [35].

A similar structure of luteolin-8-C-hexoside defined as luteolin-7-O-glucoside was reported for its antibacterial activities against* S. thyphimurium*,* P. aeruginosa*, and* S. aureus*. In that report, the antibacterial activity was reported as the zone of inhibition of the compound that could not be comparable to our MIC [36]. It is noteworthy to mention that the difference between the sugar binding to the core molecule in luteolin-7-O-glucoside is through oxygen bound (easily hydrolyzable), whereas, in our identified compound luteolin-8-C-hexoside, the binding is through a carbon, a nonhydrolysable molecule.

Lastly, antifungal activities have been reported when hyperoside was tested against a panel of fungal and bacterial phytopathogens. For example, fungal strains from the genera* Alternaria*,* Epicoccum*,* Pestalotia*,* Drechslera*, and* Fusarium* were inhibited by hyperoside at concentrations ranging from 50 to 150 *μ*g/mL [37].

Hyperoside has shown a weak antibacterial activity against* E. coli* and* S. aureus *with zone of inhibitions ranging between 0.4 and 0.9 mm [34].

Finally, the cytotoxic assessment performed in the current report showed that most of the methanolic fractions were not toxic to both macrophages and fibroblast cell lines used in this study. Another study reported that the crude extract from genetically modified hairy roots from* L. racemosa* were toxic to a panel of cell lines, including the cell lines HCT-15 (colon adenocarcinoma), Ovcar (ovarian cancer), and KB (laryngeal cancer) [38]. The main difference in the cytotoxicity when comparing to our study can be related to the different cell lines used in our study and also to the fact that we used wild plants, whereas Moreno-Anzurez' study used a genetically modified roots.

In conclusion, we report here that the methanolic fractions isolated from the leaves and flowers of* L. racemosa *showed antibacterial and antifungal activities, supporting its use in traditional medicine.

## Figures and Tables

**Table 1:** Antimicrobial activities of *L. racemosa* extracts against a panel of bacteria and fungi expressed in MIC (*μ*g/mL).

Fraction	Bacteria								Fungi			
	AB	BS	EC	MRSA	PA	SA	SAL	SE	AF	CA	CN	TM
*Leaves*												
377-382	R	R	R	R	R	R	R	500	R	R	R	R
383-396	R	R	R	R	R	R	R	500	R	R	R	R
414-417	R	R	R	R	R	R	R	R	R	R	R	250
447-506	R	R	R	R	R	R	R	R	R	R	R	125
507-516	R	R	R	R	500	R	R	R	R	R	R	R
524-525	R	R	R	R	R	R	R	500	R	R	R	250
528-533	R	R	R	R	500	R	R	500	R	R	R	R
542-547	R	R	R	R	R	R	500	R	R	R	R	R
594-603	R	R	R	R	R	R	R	R	R	R	R	500
744-749	R	R	R	500	250	500	R	R	R	R	R	R
775-784	R	R	R	R	500	500	R	R	R	R	R	R
785-794	R	R	R	R	500	R	R	R	R	R	R	500
799-810	R	R	R	R	R	R	R	R	R	R	R	500
821-833	R	R	R	R	500	R	R	R	R	R	R	R
834-844	R	R	R	R	250	R	R	R	R	R	R	R
845-851	R	R	R	R	250	R	R	R	500	R	R	R
852-856	R	R	R	500	50	500	250	500	R	R	R	R
925-941	R	R	R	R	250	R	R	R	R	R	R	R
*Flowers*												
171-180	250	500	R	R	500	R	R	R	R	R	R	R
181-190	R	R	R	R	R	R	R	500	R	R	R	R
255-262	R	R	R	R	500	R	R	R	R	R	R	R
304-310	R	R	R	R	100	R	R	R	R	R	R	R
Control	0.1^ak^	15^ak^	10^g^	10^ak^	60^g^	0.7^r^	10^ak^	1^g^	2^am^	2^am^	2^am^	1^tb^

AB, *Acinetobacter baumannii*; BS, *Bacillus subtilis*; EC, *Escherichia coli*; EF, *Enterococcus faecalis*; MRSA, methicillin-resistant *Staphylococcus aureus*; MS, *Mycobacterium smegmatis*; PA, *Pseudomonas aeruginosa*; SA, *Staphylococcus aureus*; AF, *Aspergillus fumigatus*; CA, *Candida albicans*; CN, *Cryptococcus neoformans*; TR, *Trichophyton mentagrophytes*. R, *resistant*. ^am^Amikacin, ^g^Gentamicin, ^r^Rifampicin, ^am^Amphotericin B, ^tb^ Terbinafine. Experiments were performed in triplicate.

## Data Availability

The data used to support the findings of this study are available from the corresponding author upon request.

## Abbreviations

ATCC: American type culture collection
DMSO: Dimethyl sulfoxide
ESI: Electrospray ionization
FA: Formamide
FAB: Fast atom bombardment
HPLC: High performance liquid chromatography
MeOH: Methanol
MIC: Minimum inhibitory concentration
MRSA: Methicillin-resistant* Staphylococcus aureus*
MTT: 3-(4,5-dimethylthiazol-2-yl)-2,5-diphenyltetrazolium bromide
NaOAc: Sodium acetate
NaOMe: Sodium methoxide
NMR: Nuclear Magnetic Resonance
PA: * Pseudomonas aeruginosa*
TM: * Trichophyton mentagrophytes*
UV: Ultraviolet
UV-Vis: Ultraviolet-visible.

## Supplementary Materials

Supplementary Materials 13C NMR analysis of compound 4 (hyperoside).
