# Supplementary material for: Bioactivities of Flavonoids from Lopezia racemosa
Source: Biomed Res Int. 2019 Apr 11;2019:3286489. doi: 10.1155/2019/3286489 (PMC6487151; doi:10.1155/2019/3286489)
Supplement: Supplementary Materials — 13C NMR analysis of compound 4 (hyperoside). [file 3286489.f1.pdf]

Figure S1

A

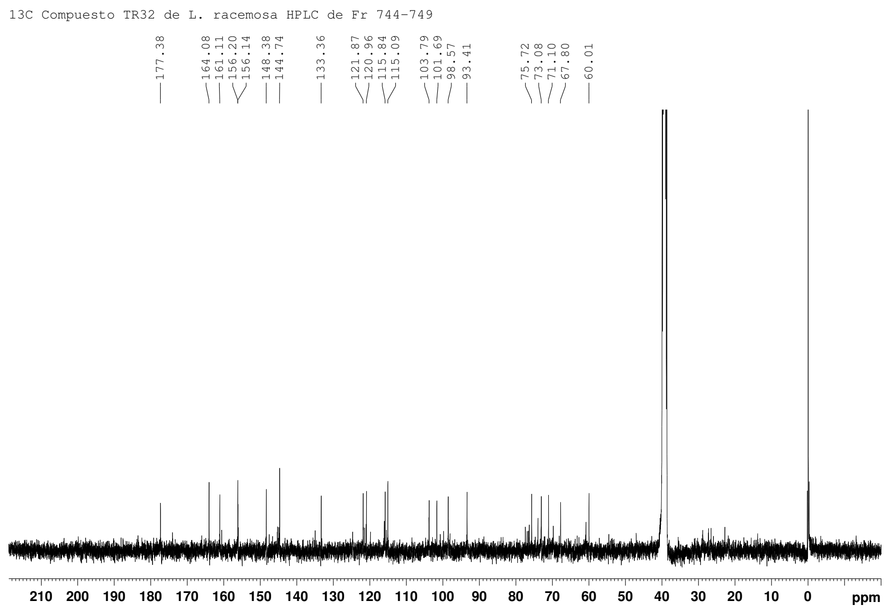

B

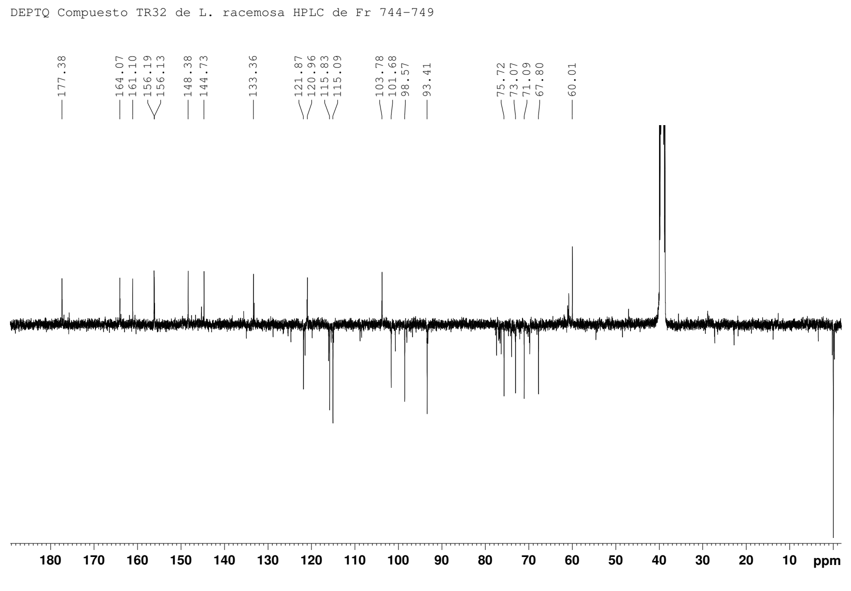

Figure S1. ^{13}C NMR analysis of compound 4 (hyperoside). The compound was analysed using (A) normal 13C NMR and (B) DEPTQ.
